# Effects of Fermentation Temperature and Aeration on Production of Natural Isoamyl Acetate by *Williopsis saturnus* var. *saturnus*

**DOI:** 10.1155/2013/870802

**Published:** 2013-06-19

**Authors:** Murat Yilmaztekin, Turgut Cabaroglu, Huseyin Erten

**Affiliations:** ^1^Inonu University, Faculty of Engineering, Department of Food Engineering, 44280 Malatya, Turkey; ^2^Cukurova University, Faculty of Agriculture, Department of Food Engineering, 01330 Adana, Turkey

## Abstract

Isoamyl acetate is a natural flavour ester, widely used as a source of banana flavour by the food industry. *Williopsis saturnus* var. *saturnus* is a yeast which can produce isoamyl acetate by esterification of amyl alcohols with acetyl coenzyme A via fermentation. The evaluation of this kind of production as an alternative way to obtain natural banana flavour could be possible, if the levels produced were high enough to make a commercial product. In this study, the effects of temperature (15°C and 25°C) and aeration (aerobic, semiaerobic, and anaerobic) on the production of isoamyl acetate by *Williopsis saturnus* var. *saturnus* from sugar beet molasses were examined. According to the results obtained, isoamyl acetate production rate and specific productivity were higher at 25°C than at 15°C and at semiaerobic condition than aerobic and anaerobic conditions. *Williopsis saturnus* var. *saturnus* showed a production rate of 0.703 mg L^−1^ h^−1^ and a specific productivity of 0.0297 mg L^−1^ cell^−1^ h^−1^ isoamyl acetate with semiaerobic condition at 25°C. The maximum amount of isoamyl acetate reached with these conditions was 118 mg/L.

## 1. Introduction

Flavour compounds found widespread application in food, beverages, cosmetics, detergents, and pharmaceutical products with a world-wide industrial size estimated at US $ 22 billion in 2012 [1]. Most of the available flavour compounds (84%) are now produced via chemical synthesis, although extraction from natural material continues [2]. However, recent market surveys have shown that consumers prefer foodstuff that can be labelled as “natural.” Although flavours may be produced by chemical transformation of natural substances, the resulting products cannot legally be labelled as natural [3]. An alternative route for flavour synthesis which is based on employment of new biotechnological processes, *de novo* microbial processes (fermentation) or bioconversions of natural precursors using microbial cells or enzymes (biocatalysis), has increased considerably in the past two decades [4–6]. 

Esters are commonly used flavouring agents, very appreciated for the fruity aromas they provide. They are employed in fruit-flavoured products, baked goods, wines, and dairy products. Acetate esters, such as ethyl acetate, hexyl acetate, isoamyl acetate, and 2-phenylethyl acetate, are recognised as important flavour compounds [3]. Especially, isoamyl acetate, the characteristic compound of banana flavour, is produced with an amount of 74 tonnes per annum [7]. Several yeasts are capable of producing large amounts of fruity ester flavours. The genus *Williopsis* synthesizes high amounts of volatile esters, for example, isoamyl acetate [8–10].

During fermentation yeasts synthesize a vast number of aroma and flavour compounds. The production of these compounds is due to yeast metabolism since significant differences in their production have been demonstrated by the use of different yeast genera, species, and strains. Apart from the choice of yeast, several factors contribute to flavour production. These include changes in fermentation conditions such as temperature, pH, aeration, agitation, and the nature and concentration of the substrate utilized [11]. It is reported that fermentation temperature, aeration, and culture medium affect the lipid metabolism, which is related to cell development, membrane integrity, and the production of several byproducts, especially those directly related to flavour compounds [12]. In this study influence of fermentation temperature and aeration on the production of isoamyl acetate by *Williopsis saturnus* var. *saturnus* (*W. saturnus*) from sugar beet molasses was investigated. 

## 2. Materials and Methods

### 2.1. Microorganism and Medium

*W. saturnus HUT 7087* was obtained from the *HUT* (Japan) culture collection. Yeast was maintained on Malt Extract Agar (0.15% Malt Extract Agar (by mass) obtained from Merck, Germany) slants and recultured monthly. Sugar beet molasses, which was obtained from the Ozmaya Co. (Adana, Turkey), was diluted with deionized water to obtain a 10 Brix molasses solution. The solution was then adjusted to pH 3.0 with 1 N H_2_SO_4_ (Merck, Darmstadt, Germany) to remove heavy metals that would affect cultivation. The molasses solution was allowed to stand for 24 h at room temperature and then centrifuged at 5000 ×g for 15 min. The supernatant was adjusted to pH 5.0 with 10 N NaOH (Merck, Darmstadt, Germany). The medium was sterilized at 121°C for 15 min in shake flasks and bioreactors and used as preculture and cultivation mediums, respectively [10].

### 2.2. Culture Conditions

Batch cultivations were carried out in duplicate 3 L laboratory bioreactors (New Brunswick, BioFlo 110, USA) containing 2 L of cultivation medium. The yeast was harvested from a 48 h preculture by centrifugation and used to inoculate the bioreactors at a level of 1 × 10^7^ cells mL^−1^. The agitation system consisted of two 6-bladed Rushton-type impellers (52 mm), operating at the stirrer speed of 100 rpm. The culture pH was left uncontrolled (pH at inoculation time was 5.0). Temperature was maintained at 15 and 25°C by a heat blanket and a chiller attached to the bioreactors for temperature experiments. A condenser was used on the top of the bioreactor to prevent the loss volatile flavour compounds. The experiments performed to examine the effect of temperature were conducted under anaerobic conditions. Aeration experiments were carried out under fully aerobic (12 L air h^−1^ = 0.2 vvm), fully anaerobic (12 L nitrogen h^−1^ = 0.2 vvm), and semiaerobic (3 L air h^−1^ = 0.05 vvm) conditions at the temperature value which was selected as best in temperature trials. Air and nitrogen were given into the bioreactors through a sterile filter during the cultivations [10]. Samples were taken at interval times from liquid medium for analytical determinations. 

### 2.3. Analytical Procedures

Parameters monitored throughout fermentations included density and viable yeast cell number. The number of viable cells was determined under a light microscope with a Thoma chamber using methylene blue staining. The supernatant of the cultivation medium was used to determine the density by a digital density meter (Mettler Toledo, Columbus, OH) [10].

The determination of products (2-methylbutanol, 3-methylbutanol, and isoamyl acetate obtained from Merck, Germany) was carried out by direct injection of samples into a gas chromatograph (Shimadzu GC-14B, Kyoto, Japan) equipped with a split/split less injector and a flame ionization detector as described by Yilmaztekin et al. [10]. Esters and higher alcohols were separated using a CP-WAX-57CB capillary column (0.25 mm i.d. × 60 m length × 0.4 *μ*m film thickness). GC settings were as follow: injection temperature: 160°C; oven temperature: 4 min at 40°C, then increased by 1.8°C per minute up to 94°C and 40°C per minute up to 180°C, and finally 4 min at 180°C; detector temperature: 180°C; carrier gas: He (1.3 mL min^−1^); split rate: 1 : 50. The quantification was performed by internal standard method. 3-Pentanol (Merck, Darmstadt, Germany) was added as an internal standard. Standard solutions containing all compounds were prepared and analysed in duplicate. Relative Response Factors (RRFs) were calculated from peak areas for each compound using,

(1) $$\begin{array}{c} \text{RRF}=\left(\frac{A_{\text{is}}}{A_{c}}\right)\times \left(\frac{C_{\text{is}}}{C_{c}}\right), \end{array}$$

where, *A*_is_ is area of internal standard, *A*_*c*_ is area of compound, *C*_is_ is concentration of internal standard, and *C*_*c*_ is concentration of compound. A linear plot was obtained with a correlation coefficient of at least 0.999 for all compounds. The results represent the means of two determinations with their standard deviations. 

### 2.4. Statistical Analysis

All experiments were carried out in duplicate independent cultures, and all results were reported as mean ± standard deviation. Data analyses were performed by using SPSS 9.0 for Windows (SPSS Inc., Chicago, IL, USA). An analysis of variance (ANOVA) was performed. A Student-Newman-Keuls test was applied on the individual variables to compare means and assess their significant differences (at significant levels of *P* ≤ 0.001 and *P* ≤ 0.05). 

## 3. Results and Discussion

The production of isoamyl acetate was influenced by the fermentative conditions, which affected the physiological activity and growth of the yeast. The amount of viable yeast cells at different fermentation conditions was given in Table 1. While low temperature and anaerobic conditions inhibited yeast growth and decreased sugar consumption, elevated temperature and aerobic fermentation conditions allow a faster and extensive yeast growth. According to Beltran et al. [12], temperature clearly affects yeast growth and fermentation kinetics. They found that fermentation rates and maximal yeast population were higher at 25°C than at 13°C in wine fermentations, but cell viability was higher at 13°C than at 25°C. This better viability of the yeasts at low temperature may be related with a different composition of the cell membrane. Furthermore, wort aeration results in increased yeast growth since oxygen is essential for key reactions in the biosynthesis of sterols and unsaturated fatty acids, which are needed for yeast growth [13]. Mauricio et al. [14] also suggested that anaerobic conditions inhibited yeast growth and decreased fructose consumption and ethanol production in wine. 

### 3.1. Effect of Temperature on Amyl Alcohols and Isoamyl Acetate Production

Isoamyl acetate synthesis by yeast is performed by the action of alcohol acetyltransferase (*AATFase*) which catalyses the reaction between amyl alcohols (2-methylbutanol and 3-methylbutanol) and acetyl-CoA. The availability of the substrates affects the amount of isoamyl acetate produced. It has been reported that the amount of isoamyl alcohol in sake mash is important on the isoamyl acetate production in sake brewing [15]. Calderbank and Hammond [16] also reported that the availability of 2-methylbutanol and 3-methylbutanol is very important in controlling the amount of isoamyl acetate. Therefore, the amount of isoamyl acetate produced is influenced by the existing concentration of amyl alcohols in the medium. 

The amount of 2-methylbutanol and 3-methylbutanol showed an increase throughout the fermentation at both 15°C and 25°C. The maximum amounts of 2-methylbutanol and 3-methylbutanol produced at 15°C were 19.2 and 12.4 mg L^−1^, respectively. The maximum concentrations of 2-methylbutanol and 3-methylbutanol at 25°C were higher than that of 15°C (24.8 and 24.9 mg L^−1^, resp.). The results demonstrated that the production of amyl alcohols was dependent on the temperature, and the higher alcohol formation was suppressed at low fermentation temperatures (15°C). Higher alcohols are produced by yeast via the catabolic (Ehrlich) and anabolic pathways (amino acid metabolism) during fermentation [17]. Yeast strain, fermentation conditions, and wort composition all have significant effects on levels of higher alcohols that are formed [18, 19]. Conditions which promote yeast growth such as high levels of nutrients (amino acids, oxygen, lipids, and zinc), increased temperature, and agitation increase the production of higher alcohols [20]. The synthesis of amyl alcohols is especially sensitive to temperature changes. Since fusel alcohols are the product of amino acid and sugar metabolism, it is recognized that parameters such as temperature affecting the assimilation of the latter also control fusel alcohol production [20]. On the other hand, conditions which restrict yeast growth—such as lower temperature and higher CO_2_ pressure—reduce the extent of higher alcohol production [21].

**Figure 1:**
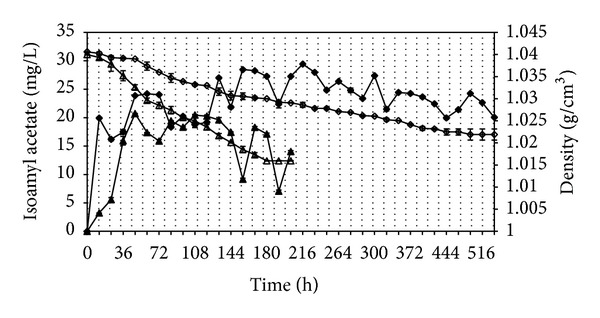
Effect of temperature on isoamyl acetate production at 15°C (*◆*) and 25°C (▲) and on density of the medium at 15°C (*◊*) and 25°C (Δ). The bars indicate standard deviations for two independent cultivations.

The effect of temperature on isoamyl acetate production patterns by *Williopsis saturnus* var. *saturnus* is shown in Figure 1 and Table 2, respectively. As can be seen in Figure 1, there was a slow decrease in the density of the medium at 15°C compared to 25°C. However, the amount of isoamyl acetate produced against the consumed substrate was higher at 15°C. The concentration, production rate, and specific productivity of isoamyl acetate and ester/higher alcohol ratio at 15°C and 25°C were significantly different (*P* ≤ 0.05). While the maximum isoamyl acetate produced was higher at experiment performed 15°C (29.4 mg L^−1^) than at 25°C (20.7 mg L^−1^), on the contrary isoamyl acetate production rate was higher at 25°C (0.43 mg L^−1^ h^−1^) than at 15°C (0.14 mg L^−1^ h^−1^). The specific productivity was also higher at 25°C (0.044 mg L^−1^ cell^−1^ h^−1^) compared to 15°C (0.024 mg L^−1^ cell^−1^ h^−1^). The ratio of the isoamyl acetate to total amyl alcohols, which is a parameter known as to influence the sensory properties of the medium, was also influenced by temperature. An increased content of esters gives an enhanced fruity flavour, and this can improve when higher alcohol content decreases [10]. 

Esters are the product of an enzyme-catalyzed condensation reaction between acyl-CoA and a higher alcohol [22]. Fundamentally, two factors are important for the rate of ester formation during fermentation: the availability of the two substrates (acetyl/acyl-CoA and alcohols) and the activity of enzymes (*AATFase*). Hence, all parameters that affect substrate concentrations or enzyme activity will influence ester production [13, 19, 23, 24]. Dufour et al. [23] indicated that acetate esters increased with temperature during fermentation, and this increase is related to the stimulation of the *AATases*. Furthermore, it is well known that the formation of higher alcohols is also temperature dependent, so that changes in temperature may cause changes in the availability of fusel alcohols that are necessary for ester formation [16]. Generally, increased fermentation temperatures in the range of 10–25°C lead to increased ester production. According to Peddie [13], when temperature is elevated, the concentration of esters produced during the fermentation is also increased due to increasing membrane fluidity. An increase in membrane fluidity may allow more esters to diffuse into the medium. Verstrepen et al. [22] reported that up to 75% more esters are produced at 12°C than at 10°C. However, different esters may show different temperature dependencies. Some studies show that ethyl acetate and phenyl ethyl acetate are produced maximally at 20°C, whereas maximal production of isoamyl acetate and ethyl caprylate occurs at 15°C [18]. This temperature dependency is not valid for all yeast strains, and some strains may show different temperature dependencies for the production of certain volatile esters [25]. In this study, the production rate and specific productivity parameters were taken into account for the performance of temperature trials. Hence, the subsequent trials to investigate the effect of aeration on isoamyl acetate production were carried out at 25°C. 

### 3.2. Effect of Aeration on Amyl Alcohols and Isoamyl Acetate Production

In the course of anaerobic fermentation, the production of amyl alcohols was inhibited compared to semiaerobic and aerobic conditions. The maximum concentrations of 2-methylbutanol and 3-methylbutanol were obtained with semiaerobic conditions (92.8 mg L^−1^ and 43.8 mg L^−1^, resp.) followed by aerobic and anaerobic fermentations. Under semiaerobic conditions, the concentrations of amyl alcohols peaked at exponential phase and then decreased at stationary phase, probably as a result of their utilization for isoamyl acetate production. Results showed that aeration stimulates the production of amyl alcohols.

Brányik et al. [26] stated that control of higher alcohol formation can be well balanced by the choice of an appropriate yeast strain, wort composition (amino acids, lipids, zinc), fermentation conditions (temperature, aeration, and residence time), immobilization method, and reactor design. The majority of these interventions are based on the stimulation of the growth intensity, for example, dissolved oxygen concentration and temperature, enhancing the higher alcohol formation. According to Crowel and Guymon [27], aerated fermentations formed as much as five times the amount formed under anaerobic conditions and the amyl alcohols accounted for most of the increase resulting from aerobic conditions. Some authors also demonstrated that aeration stimulated the production of higher alcohols [19, 24, 28–30].

**Figure 2:**
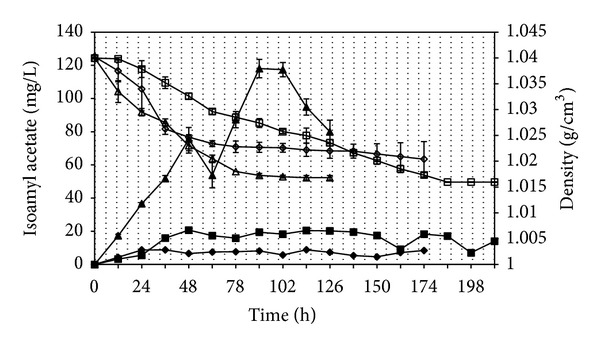
Effect of aeration on isoamyl acetate production at aerobic (*◆*), anaerobic (■) and semiaerobic (▲) conditions and on density of the medium at aerobic (*◊*), anaerobic (□), and semiaerobic (Δ) conditions. The bars indicate standard deviations for two independent cultivations.

The effect of aeration on substrate consumption and isoamyl acetate production by *Williopsis saturnus *var. *saturnus* is shown in Figure 2. There was a sharp decrease with the density of the medium at semiaerobic experiment compared to aerobic and unaerobic experiments which is related to yeast growth. The isoamyl acetate production rate, specific productivity, and ester/higher alcohol ratio were given in Table 3. The concentration, production rate, and specific productivity of isoamyl acetate were significantly affected with aeration conditions (*P* ≤ 0.05). The maximum isoamyl acetate concentration was observed with semiaerobic condition (118.1 mg L^−1^), followed by anaerobic and aerobic fermentations (20.7 and 8.8 mg L^−1^, resp.).

Mauricio et al. [14] reported that the addition of oxygen to the must increases ethyl acetate and fatty acid ethyl esters concentrations, decreasing those of higher alcohols, 2,3-butandiol, acetic acid, and higher alcohol acetates. Contrary to the mentioned results, Inoue et al. [15] demonstrated that *Hansenula mrakii* could produce a large amount of isoamyl acetate when the cells were cultured under aerobic conditions. According to Peddie [13], wort aeration results in increased yeast growth since oxygen is essential for key interactions in the biosynthesis of sterols and unsaturated fatty acids, which are needed for yeast growth. Increased yeast growth creates additional demand for acetyl CoA for that purpose, and so the availability of acetyl CoA for ester synthesis is reduced; even a low rate of aeration during fermentations can strongly inhibit ester formation [13, 18, 19, 24, 31]. Generally, anaerobic conditions decreased the production of butyl acetate, isoamyl acetate, phenethyl acetate, and hexyl acetate, compared to semiaerobic conditions [14]. Under semiaerobic conditions, the concentrations of the acetates peaked and then decreased, probably as a result of their hydrolysis under the action of cellular esterases, the activity of which increases at the end of fermentation [32]. Confirming these results, in our study the concentration of isoamyl acetate peaked at the end of cellular growth under semiaerobic conditions and then decreased at the end of fermentation. 

## 4. Conclusions 

Temperatures studied had a significant effect on the production of isoamyl acetate by *Williopsis saturnus* var. *saturnus *using sugar beet molasses. The maximum isoamyl acetate concentration was reached at 15°C, while the maximum isoamyl acetate production rate was at 25°C. Aeration was an important factor for the production of isoamyl acetate. Isoamyl acetate concentration showed a decrease at aerobic and anaerobic conditions. At certain levels of aeration (semiaerobic) isoamyl acetate concentration was at maximum. These results showed that the production of isoamyl acetate by *Williopsis saturnus* var. *saturnus* appears to be a potential way for isoamyl acetate production. However, an amount of 118 mg/L is still not enough to make it commercial. Further studies are required regarding genetic modifications on yeast strain as well as suitable bioprocessing strategies for an efficient isoamyl acetate production process.

## Figures and Tables

**Table 1:** Viable yeast cells (×10^7^ cells mL^−1^) at different fermentation conditions.

Fermentation conditions	Time (h)						
	0	36	72	108	144	180	216
15°C	1.0 ± 0.12	2.9 ± 0.14	4.65 ± 0.10	6.25 ± 0.17	6.65 ± 0.20	7.25 ± 0.23	5.8 ± 0.11
25°C	1.0 ± 0.22	7.18 ± 0.04	10.3 ± 0.35	8.95 ± 0.10	10.2 ± 0.10	9.35 ± 0.21	5.58 ± 0.17
Anaerobic	1.0 ± 0.24	7.35 ± 0.31	10.3 ± 0.44	10.2 ± 0.34	10.45 ± 0.10	8.58 ± 0.22	NT*
Semi-aerobic	1.0 ± 0.18	17.5 ± 0.54	28.4 ± 0.49	24.6 ± 0.44	25.8 ± 0.51	24.6 ± 0.33	23.6 ± 0.21
Aerobic	1.0 ± 0.16	21.75 ± 0.35	20 ± 0.15	22.1 ± 0.13	20 ± 0.22	NT*	NT*

*NT: not tested.

**Table 2:** Effect of temperature on isoamyl acetate concentration, production rate, specific productivity, and ester/higher alcohol ratio.

Temperature	Isoamyl acetate (mg L^−1^)	Isoamyl acetate production rate^x^ (mg L^−1^ h^−1^)	Isoamyl acetate specific productivity^x^ (mg L^−1^ cell^−1^ h^−1^)	Isoamyl acetate/total amyl alcohol ratio^x^
15°C	29.43 ± 0.10	0.136 ± 0.0005	0.0235 ± 0.0002	1.75 ± 0.23
25°C	20.72 ± 0.02	0.432 ± 0.0004	0.0436 ± 0.0003	0.69 ± 0.12
Level of significance	∗∗	∗∗	∗∗	∗∗

^x^Isoamyl acetate production rate, specific productivity, and ester/higher alcohol ratio were calculated at times when isoamyl acetate concentration reach the maximum. **: *P* ≤ 0.001.

**Table 3:** Effect of aeration on isoamyl acetate concentration, production rate, and specific productivity.

Aeration	Isoamyl acetate (mg L^−1^)	Isoamyl acetate production rate^x^ (mg L^−1^ h^−1^)	Isoamyl acetate specific productivity^x^ (mg L^−1^ cell^−1^ h^−1^)	Isoamyl acetate/total amyl alcohol ratio^x^
Aerobic	8.84 ± 0.08^c^	0.082 ± 0.0005^c^	0.0037 ± 0.0003^c^	1.71 ± 0.32^cy^
Anaerobic	20.72 ± 0.02^b^	0.432 ± 0.0004^b^	0.0276 ± 0.0011^b^	0.77 ± 0.02^b^
Semiaerobic	118.05 ± 5.59^a^	0.703 ± 0.0333^a^	0.0297 ± 0.0009^a^	1.37 ± 0.60^a^
Level of significance	∗	∗	∗	∗

^x^Isoamyl acetate production rate, specific productivity, and ester/higher alcohol ratio were at times when isoamyl acetate concentration reach the maximum. ^y^Different letters in the same column indicate significant difference (*P* ≤ 0.05). ∗: *P* ≤ 0.001.
